# Intraoperative Kirschner Wire Migration during Robotic Minimally Invasive Spine Surgery

**DOI:** 10.1155/2019/9581285

**Published:** 2019-11-24

**Authors:** Ashley Peterson, Lynn K. Ngai, Mark A. Burbridge

**Affiliations:** Department of Anesthesiology, Perioperative and Pain Medicine, Stanford University School of Medicine, Stanford, CA, USA

## Abstract

We present the case of a 58-year-old woman who underwent a minimally invasive robotic-assisted L4-S1 instrumentation and fusion which was complicated by a Kirschner wire (K-wire) fracture and migration into the abdominal cavity necessitating emergent exploratory laparotomy. Retrieval of the K-wire proceeded without incident, and the patient had an otherwise uneventful surgery and recovery. This is the first such case description reported in the literature. As minimally invasive robotic-assisted spine procedures become more common, it is essential for the anesthesiologist to be familiar with potential complications to manage such patients in the perioperative period optimally.

## 1. Introduction

Spine surgery is a commonly encountered procedure for the anesthesiologist. Common complications of spine surgery involve the potential for significant blood loss, as well as those related to prone positioning [1]. Injuries to organs in close proximity to the spine have also been reported, and include the heart [2], lungs [3], aorta [4], spleen [5], liver [6], and bowel [7] to name a few. Recent advances in robotic technology have resulted in an increasing number of these procedures with a minimally invasive approach utilizing robotic technology, which is reported to have increased accuracy compared to traditional open spine procedures. While there are potential advantages of such an approach, we present for the first time the case of a Kirschner wire (K-wire) that fractured during placement and subsequently migrated into the abdominal cavity necessitating emergent exploratory laparotomy. This rare complication may become more common as the numbers of minimally invasive spine surgery continue to increase. This patient provided a written consent for this case report.

## 2. Case Presentation

This patient was a 58-year-old female with a body mass index (BMI) of 46. She had no other significant medical problems or allergies. Her medical history was positive for medically refractory back and leg pain. The proposed surgery was a minimally invasive robotic-assisted posterior L4-S1 instrumentation and fusion. This patient had this surgery performed as part of a staged procedure; she had an L4-S1 anterior lumbar interbody fusion performed the previous day by way of laparotomy, and tolerated this very well without surgical complications. The indication for a minimally invasive robotic-assisted approach was to minimize postoperative pain to promote a faster recovery due to her high BMI and deconditioned physical status.

**Figure 1:**
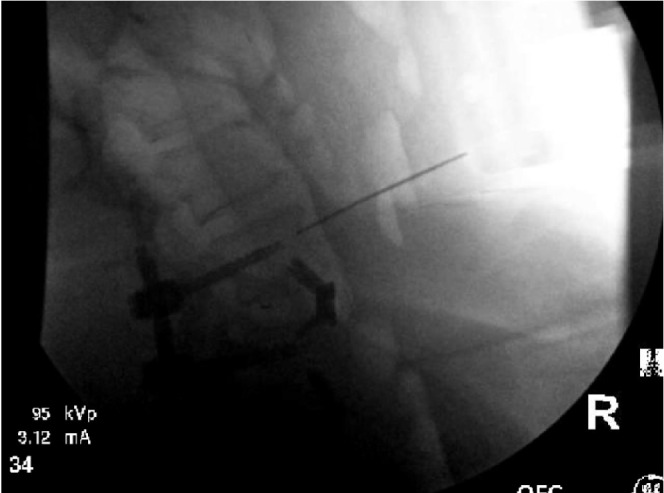
Kirschner wire projecting anteriorly from L5.

Approximately 2 hours into the procedure, during the passage of the 1.3 mm, 25 centimeter long stainless steel Kirschner wire into the second surgically approached vertebral body (L5), a sudden loss of resistance was noted by the surgeon. The surgeon estimated that the K-wire passed at least 6−8 cm too deep. When an attempt was made to pull the K-wire out of the pedicle, it completely snapped, with the broken edge deeply recessed within the bone. Attempts were made to curette the bone to access the wire so that it could be retrieved, but this was not possible. An intraoperative radiograph showed the K-wire projecting anteriorly from the body of the L5 vertebrae (Figure 1). No changes in vital signs were noted at this time, but a high index of suspicion was immediately noted for major vascular (such as the aorta or inferior vena cava) or visceral (such as bowel or liver) injury. An arterial line had been inserted after induction, but at this time a second 14 gauge IV catheter was inserted, and four units of blood were emergently transported to the OR along with a Level-1 rapid infusion system.

A discussion then took place between the neurosurgery, vascular surgery, and anesthesia teams. While an emergent CT scan would have been useful to determine the precise location of the K-wire, it was felt that with the unknown location of the wire, and the risk of the patient becoming unstable during transport due to the unknown nature of the injury and location of the K-wire, that the risk of this approach was prohibitive. Instead, a portable intraoperative CT scanner was utilized but did not add much visual definition pertaining to the location of the K-wire. The more imminent threat; however, was the risk of vascular or visceral injury when the patient was turned from prone to supine for the exploratory laparotomy to remove the wire and repair any injuries that may have been sustained.

Upon repositioning the patient to the supine position, no significant changes in vital signs occurred. The exploratory laparotomy proceeded and became apparent that the K-wire was no longer retained in the L5 vertebral body, but was not found in the retroperitoneal space either. It was not until the intraabdominal exploration that the 8 cm long fragment of the K-wire was found within some loops of the small bowel. A subsequent intraoperative radiograph confirmed no other fragments of the K-wire were retained in the abdominal cavity. A full exploratory laparotomy was then performed, and a complete run of the bowel did not reveal any injuries. A primary concern was that if a bowel injury had occurred, it would have compromised the sterility of the surgical implants used in the previous spinal instrumentation necessitating its removal; however, this was not the case. In retrospect, it was felt that a shorter K-wire might have allowed more precise control over the depth it was advanced, mainly if less force was applied.

## 3. Discussion

The Mazor Robotics Renaissance^™^ Guidance System is a minimally invasive spine surgery system that utilizes preoperative imaging to determine optimal pedicle screw trajectory by way of a percutaneous approach. This technology is reported to provide improved accuracy of pedicle screws, smaller incisions, and scars, less scar tissue around the spine, less blood loss, less pain after surgery and therefore less pain medicine, and faster recovery after surgery. A robotic arm determines the optimal trajectory and serves as a drill guide for the surgical team. At each site, a skin incision is made, then a drill is used to make a pilot hole in the pedicle, a K-wire is drilled, and then the cannulated pedicle screw is passed over the K-wire into the pedicle and tightened. When all pedicle screws are fastened, a rod is placed through the existing incisions, and then the rod is tightened down [8].

Complications associated with minimally invasive robotic spine surgery are sparse. A review of one year of spine surgeries utilizing robotic assistance for pedicle screw placement documented two cases in which K-wires were dislodged and required reinsertion; however, no mention was made of structures at risk from the dislodged K-wires or additional exploratory procedures required to retrieve the dislodged K wires [9]. In reporting this case, we documented the occurrence of the K-wire migration due to direct mechanical force during the use of robotic-assisted spine surgery.

K-wires have; however, migrated during other types of surgery. When left in place in fracture fixation procedures, spontaneous K-wire migrations have occurred weeks to years after placement. K-wires used in fixation of the clavicle have migrated to the soft tissues of the head and neck, the lungs, and even into the spine [10]. When used in hip and pelvic fracture fixations, K-wires have migrated within the pelvis causing soft tissue damage, fistulous tracts between the external iliac artery and the appendix [11], and even long-distance migration to the right ventricle of the heart [12]. To prevent this complication, it is recommended that the wires to be left visible outside the skin when possible, and their ends bent when direct visualization is not possible, and be removed when no longer needed. A single case report of intraoperative K-wire migration due to direct mechanical force with a real risk of compromise to multiple vascular and soft tissue structures requiring acute laparotomy has been documented following revision total hip arthroplasty [13].

## 4. Summary

We report for the first time the anesthetic management of a K-wire that migrated beyond the anterior body of the L5 vertebrae into the peritoneal cavity with a real risk of damage to major vascular and soft tissue structures requiring emergent exploratory laparotomy. The anesthesiologist should be aware of this potential complication as the number of robotic-assisted minimally invasive spine surgical procedures to increase in number.
